# HOTAIR regulates HK2 expression by binding endogenous miR-125 and miR-143 in oesophageal squamous cell carcinoma progression

**DOI:** 10.18632/oncotarget.21195

**Published:** 2017-09-23

**Authors:** Jian Ma, Yanxin Fan, Tingting Feng, Fangjun Chen, Zhipeng Xu, Suqing Li, Qingfeng Lin, Xiaoting He, Weihong Shi, Yang Liu, Xiufeng Cao, Bin Zhu, Zhihua Liu

**Affiliations:** ^1^ Department of Surgical Oncology, Affiliated Nanjing First Hospital of Nanjing Medical University, Oncology Center of Nanjing Medical University, Nanjing 210008, Jiangsu Province, China; ^2^ Jiangsu Cancer Hospital, Medical School of Nanjing University, Nanjing 210008, Jiangsu Province, China; ^3^ Institute of Biology and Medical Sciences, Jiangsu Key Laboratory of Infection and Immunity, Soochow University, Suzhou 215123, Jiangsu Province, China; ^4^ The Comprehensive Cancer Centre of Drum Tower Hospital, Medical School of Nanjing University, Clinical Cancer Institute of Nanjing University, Nanjing 210008, Jiangsu Province, China; ^5^ Yancheng Vocational Institute of Health Sciences, Yancheng 224005, Jiangsu Province, China; ^6^ Department of Pharmacy, No.401 Hospital of Chinese People's Liberation Army, Qingdao 266071, Shandong Province, China; ^7^ State Key Laboratory of Molecular Oncology, National Cancer Center/Cancer Hospital, Chinese Academy of Medical Sciences, Beijing 100021, China

**Keywords:** HOTAIR, miR-125, miR-143, ESCC, HK2

## Abstract

Esophageal Squamous Cell Carcinoma (ESCC) is one of the most common malignant cancers worldwide with a high death rate worldwide. Long non-coding RNA (LncRNA) has been recently demonstrated to play a critical role in ESCC. LncRNA HOTAIR played important regulatory roles in ESCC. We highlight the molecular mechanisms by which HOTAIR could influence the expression of Hexokinase 2 (HK2) in ESCC through binding miR-125 and miR-143 directly. Taken together, this study identified a functional lncRNA HOTAIR involved with regulation of glycolysis via miRNA-125/miRNA-143-HK2 in ESCC cells. The “competitive endogenous RNA” (ceRNA) model of HOTAIR/miR-125 and miR143/HK2 interaction might serve as important targets for ESCC diagnosis and therapy.

## INTRODUCTION

Oesophageal cancer is the 8th most common cancer and the 6th leading cause of cancer-related death worldwide [1]. The incidence of oesophageal adenocarcinoma in Western countries is increasing, and the predominant type of oesophageal cancer is squamous cell carcinoma [2]. In Asia, 90% of oesophageal cancer has the histological type of ESCC, and China is one of the high-risk oesophageal cancer regions [3]. Because diagnosis is rarely made prior to advanced disease stages, the overall 5-year survival rate remains less than 20% [4–6]. To improve outcomes in this prevalent form of cancer, new molecular markers for early detection and prognostic analysis are urgently required.

In malignant cells, glucose is preferentially metabolized through aerobic glycolysis, and oxidative phosphorylation in mitochondria is often decreased compared to normal cells. This phenomenon, termed the aerobic glycolysis effect or the Warburg effect, is characterized by increased glycolysis and lactate production regardless of oxygen availability [7]. To acquire this high glycolytic phenotype (known as the Warburg effect) cancer cells deregulate the expression and/or activity of some crucial molecular factors in glucose metabolism, such as hexokinase enzymes that catalyse the first and irreversible step of the glycolytic pathway by converting glucose into glucose-6-phosphate [8–10]. In humans, there are four known hexokinase isoforms with specific biochemical properties [11]. Hexokinase 2 (HK2) is the predominant isozyme that is overexpressed in tumours and contributes to aerobic glycolysis. Thus, it is documented as a pivotal player in the Warburg effect and has been proposed as a metabolic target for cancer therapy [10, 12]. The relationships between oncogenes or tumour suppressor genes and HK2 expression and the mechanism of these interactions have rarely been reported with regard to the development of ESCC.

LncRNAs are mRNA-like transcripts that range in length from 200 nt to ∼100 kilobases (kb) and lack significant open reading frames. They have no protein coding potential but play an important role in regulating cell differentiation and development [13, 14], gene transcription and translation [15, 16], genetic and epigenetic regulation [17, 18] and other cell activities. Their dysregulation has been found in various types of carcinomas, including melanoma [19], breast cancer [20], hepatocellular carcinoma [21], colon cancer [22], prostate cancer [23], lung cancer [24], and bladder cancer [25]. The regulatory mechanism of lncRNAs on a genome wide scale is not well understood [26]. The hypothesis of ceRNA states that all types of RNA transcripts (messenger RNAs, transcribed pseudogenes, and lncRNAs) communicate through microRNA [27]. The notion of endogenous lncRNA sponges was recently linked to the progression of liver cancer [28].

HOTAIR, HOX transcript antisense RNA, is capable of reprogramming chromatin organization and promoting cancer cell metastasis [29, 30]. We have previously reported that the upregulation of HOTAIR promotes ESCC metastasis and indicates a poor prognosis [31]. However, its mechanism in ESCC remains unclear and needs in-depth investigation.

Based on the ceRNA hypothesis, the HOTAIR and miRNA may have a very close interaction. This interaction possibly plays an important regulatory role in the development of ESCC and could bring new clues for exploring the molecular mechanisms of oesophageal carcinogenesis.

We predicted and screened potential miRNA targets of HOTAIR using Refseq and bioinformatics software. Subsequently, dual-luciferase reporter assays demonstrated microRNA-125 (miR-125) and microRNA-143 (miR-143) binding to HOTAIR. Furthermore, a mechanistic analysis revealed that HOTAIR positively regulates HK2 expression by sponging miR-125 and miR-143, thus playing an oncogenic role in ESCC.

## RESULTS

### HOTAIR promoted cell proliferation *in vitro* and *in vivo*

**Figure 1:**
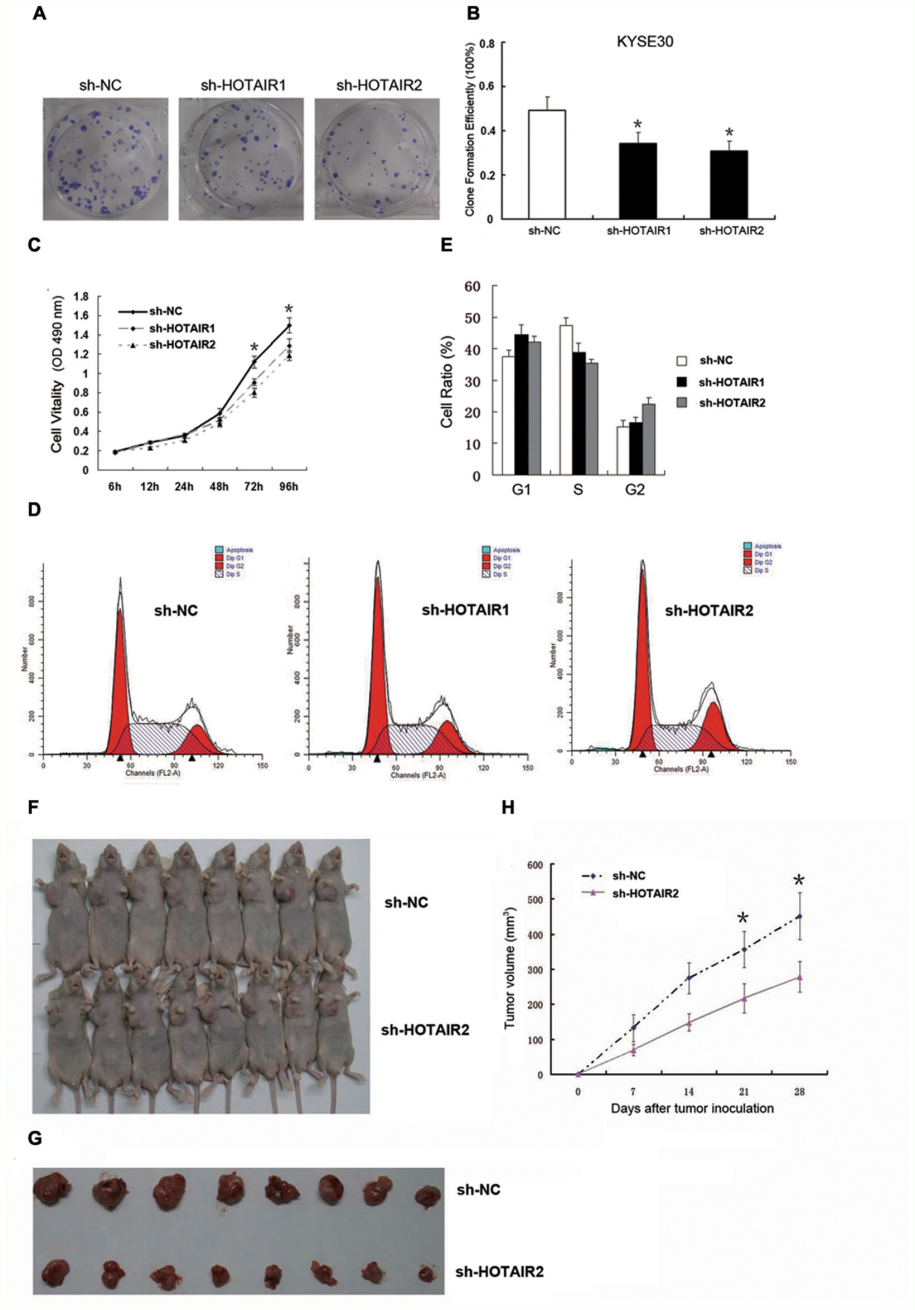
HOTAIR promoted cell proliferation *in vitro* and *in vivo* **(A, B)** Clone formation ability in the KYSE30 cells. **(C)** Cell proliferation in the KYSE30 cells was determined by CCK-8 assay. **(D, E)** Cell cycle distribution in the KYSE30 cells was determined by flow cytometry using propidium iodide (PI) staining. **(F)** Male BALB/c nude mice were injected subcutaneously into the right flank with transfected KYSE30 cells, and tumours were allowed to grow for 4 weeks to establish a subcutaneously and orthotopically implanted mouse model. **(G)** Representative picture of tumour formation of xenografts in nude mice in the sh-NC and sh-HOTAIR groups (each group *n*=8). **(H)** Summary of tumour volumes in mice in the sh-NC and sh-HOTAIR groups; measurements were obtained every week. Data are shown as the mean±standard deviation (SD). *p < 0.05. Representative results from at least 3 independent experiments.

Malignant proliferation is a well-known and critical cellular process of cancer cells. The HOTAIR expression level was determined to be a powerful independent prognostic indicator for ESCC [31]. To explore whether the level of HOTAIR expression affects tumourigenesis, colony formation assays were conducted to detect cell growth viability in RNA interference (RNAi) treated KYSE30 cells. First, RNAi approach was employed to knock down endogenous HOTAIR in KYSE30 cells. After incubating cells at 37°C and 5% CO_2_ for 14 days, we found that depletion of HOTAIR resulted in decreased cell proliferation compared with negative control (shRNA-NC, sh-NC) (Figure 1A). The colonies were counted, and the clone formation efficiency of the sh-HOTAIR1 group was 34.25% and the sh-HOTAIR2 group was 30.75%, while the control group was 49.25% (*p* < 0.05, Figure 1B). Significant inhibition of KYSE30 cell growth was observed after depletion of HOTAIR expression after 96 h, indicating that HOTAIR plays an essential role in the control of cancer cell growth. The CCK-8 assay was used to examine whether HOTAIR regulates cell cycle progression. Knockdown of HOTAIR led to a change in cell cycle distribution (*p* < 0.05, Figure 1C). The fraction of KYSE30 cells in G1 phase increased from 38.75% (sh-NC) to 44.47% (shRNA-HOTAIR1, sh-HOTAIR1) and 42.15% (shRNA-HOTAIR2, sh-HOTAIR2), while the fraction of cells in S phase decreased from 38.52.% (sh-NC) to 35.40% (sh-HOTAIR2); the fraction of cells in S phase in the sh-HOTAIR1 group was not changed (38.42.%), but the fraction of cells in G2-M phase decreased from 22.73% to 16.62% (sh-HOTAIR1) (Figure 1D and 1E). The portion of the cell population in G1 phase was increased but that in S phase or G2 phase was reduced after depletion of HOTAIR compared with cells transfected with sh-NC, suggesting that HOTAIR may affect the G1/S transition. The cell cycle transition was also consistent with the finding that HOTAIR promotes ESCC cell proliferation.

Furthermore, sh-HOTAIR2/sh-NC-transfected KYSE30 cells were inoculated into male nude mice. Four weeks after injection, the tumours formed in the sh-NC group were substantially bigger than those in the sh-HOTAIR2 group (Figure 1F and 1H). Moreover, the tumour volume at the end of the experiment was markedly higher in the sh-NC group (451.52±66.78 mm^3^) than in the sh-HOTAIR2 group (278.43±43.12 mm^3^; Figure 1G). These results indicated that inhibition of HOTAIR expression could suppress tumour growth *in vitro* and *in vivo*.

### Identification of potential HOTAIR-targeting miRNAs

**Figure 2:**
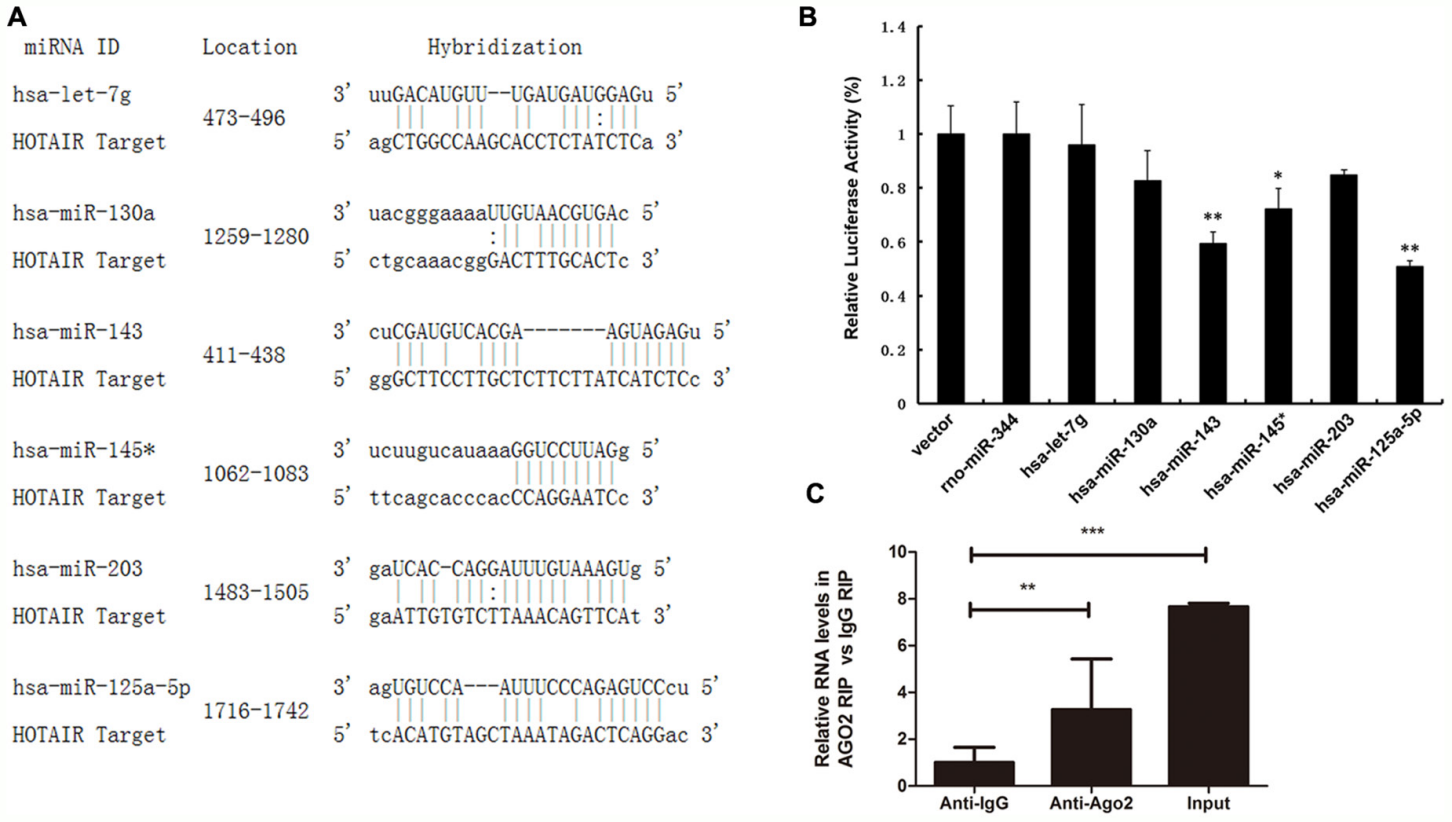
Identification of potential HOTAIR-targeting miRNAs **(A)** Schematic representation of the binding sites between the miRNAs and HOTAIR. **(B)** Luciferase activity in HEK293T cells cotransfected with miR-let7g/130a/143/145*/203/125 mimics and luciferase reporters containing control vector and HOTAIR. **(C)** The relative RNA level was detected in the substrate of a RIP assay by qRT-PCR. Triplicate experiments were analysed, and the mean±SD is shown; *p < 0.05, **p < 0.01, ***p < 0.001.

Evidence from both mammalian and plant systems support the existence of endogenous mechanisms of miRNA titration, whereby mRNAs, pseudogenes and long noncoding RNAs compete for miRNA binding [8, 32]. Very recently, the repertoire of ceRNA was expanded by the identification of a new class of circular RNAs [33, 34]. We wanted to know the molecular mechanisms and molecular targets regulated by HOTAIR in ESCC. Prediction of miRNA target sites was performed using the online software RegRNA 2.0 (http://regrna2.mbc.nctu.edu.tw/detection.html). We noted 564 target miRNAs, and among them, 48 miRNAs were predicted by both RegRNA 2.0 and Targetscan. Of these candidate 48 miRNAs targeting HOTAIR (data not shown), 6 miRNAs (miR-let7g/130a/143/145*/203/125a-5p) have putative binding sites for HOTAIR (Figure 2A).

Meanwhile, luciferase reporter assays were performed to confirm the binding of miRNAs to HOTAIR. pLUC-HOTAIR was co-transfected with pLMP-hsa-miRNA or empty pLMP plasmid (as a control) into HEK293T cells, with rno-miR-344 as a negative control. As expected, luciferase activities were reduced with respect to the control plasmid (pCtrl) when all the chosen miRNAs were expressed. In particular, the luciferase activities of miR-125 and miR-143 were decreased by 50.94% and 59.38% (Figure 2B).

miRNA- and siRNA-guided Argonaute proteins (Ago proteins including Ago2) silence mRNA expression through the RNA-Induced Silencing Complex (RISC) [35–37]. RNA immunoprecipitation (RIP) was conducted to explore the interaction between HOTAIR and Ago2. HOTAIR was preferentially enriched in Ago2-containing miRNPs relative to control immunoglobulin G (IgG) immunoprecipitates (Figure 2C). This finding suggested that HOTAIR can bind miR-125 and miR-143 and regulate their expression, consistent with our bioinformatic analysis and luciferase assays.

### miR-125 and miR143 post-transcriptionally inhibit HK2 in ESCC

**Figure 3:**
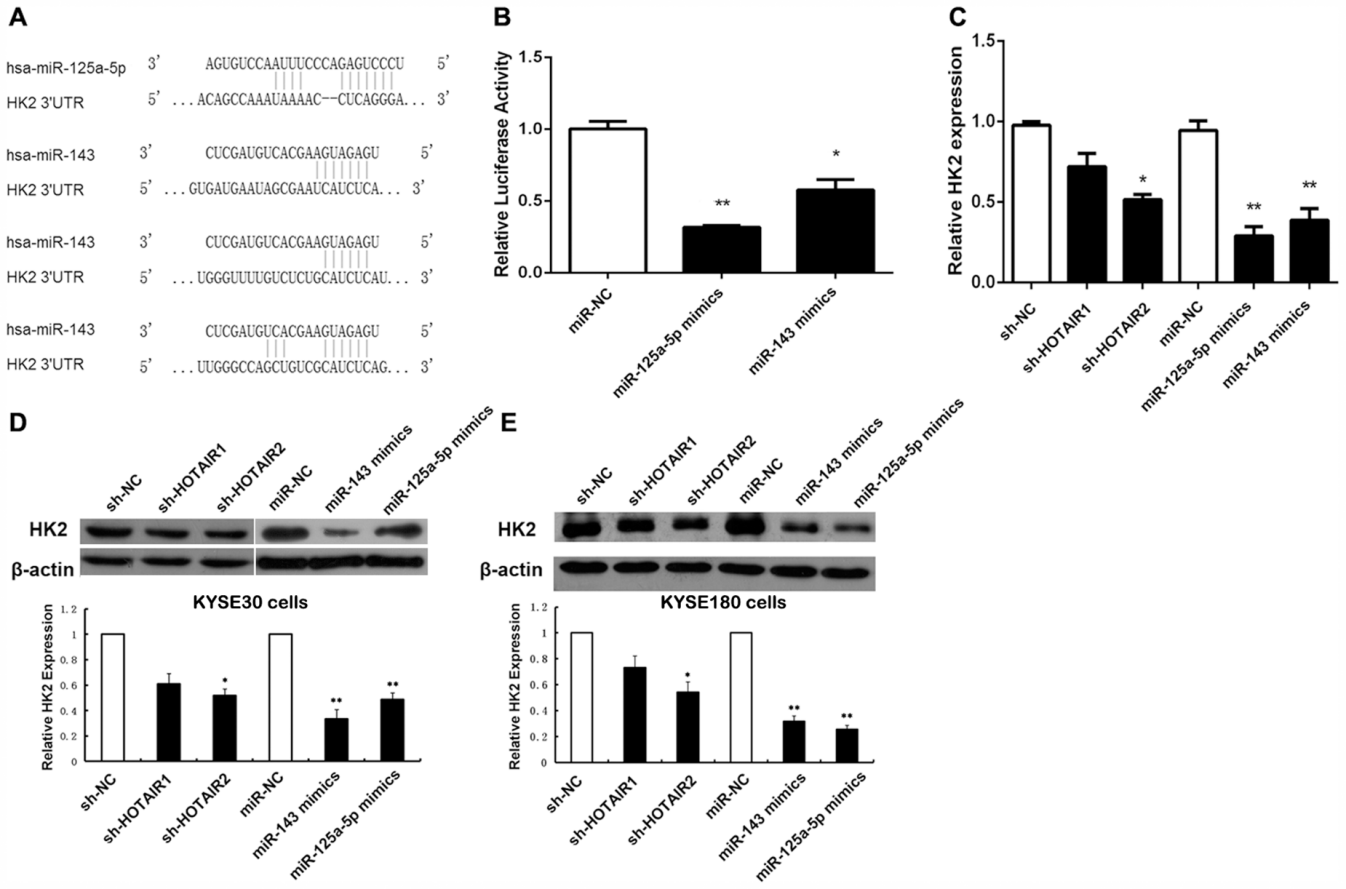
HOTAIR regulates HK2 expression via miR-125a-5p/143 **(A)** Schematic representation of the binding sites between miRNAs and HK2. **(B)** Luciferase activity in HEK293T cells cotransfected with miR-125a-5p and miR-143 mimics and luciferase reporters containing control vector and HK2. **(C)** The mRNA levels of HK2 in ESCC cells transfected with sh-NC, sh-HOTAIR, miR-NC, miR-125a-5p, and miR-143. **(D, E)** Protein expression level of HK2 in KYSE30 ESCC cells (D) and KYSE180 cells (E) transfected with sh-NC, sh-HOTAIR, miR-NC, miR-125a-5p, and miR-143. Data are shown as the mean±standard deviation (SD). *p < 0.05, **p < 0.01. Representative results from at least 3 independent experiments.

HK2 plays essential roles in tumour growth, survival, and metastasis [38]. To further prove these in ESCC, we used Target Scan and Pictar bioinformatics tools to search for miRNA binding sites in the 3'UTR of HK2. We found that the 3'UTR of HK2 contains 1 miR-125 binding site and 3 miR-143 binding sites (Figure 3A).

To demonstrate a direct effect of miR-125 and miR-143 on HK2 mRNA, we cloned the 3'UTR of human HK2 mRNA into the 3'UTR of a luciferase reporter and measured the luciferase activity. The expression of miR-125 and miR-143 dramatically reduced the luciferase activity in 293T cells (Figure 3B). Consequently, we decided to further analyse the miR-125/miR-143 and HK2 connection. We overexpressed miR-125 and miR-143 separately in KYSE30 cells and KYSE180 cells to analyse the expression levels of HK2. We found that ectopic expression of miR-125 and miR-143 induced a marked reduction in HK2, both at the mRNA and protein level, in KYSE30 cells (Figure 3C and 3D) and KYSE180 cells (Figure 3E). These data demonstrate that miR-125 and miR-143 control HK2 expression by targeting the 3'UTR of HK2.

### HOTAIR regulates HK2 expression via miR-125/143

Because miR-125 and miR-143 could be regulated by HOTAIR in ESCC and HK2 was found to be a target gene of miR-125 and miR-143, we examined the expression level of HK2 during HOTAIR depletion. As shown in Figure 3C, the transcription levels of HK2 decreased by approximately 51% after HOTAIR depletion. Western blotting was performed to address the role of HOTAIR in HK2 expression (Figure 3D and 3E). The expression of HK2 was significantly inhibited during HOTAIR RNAi, consistent with the transcription level results.

### HK2 promoted cell proliferation, invasion and migration in ESCC cells

**Figure 4:**
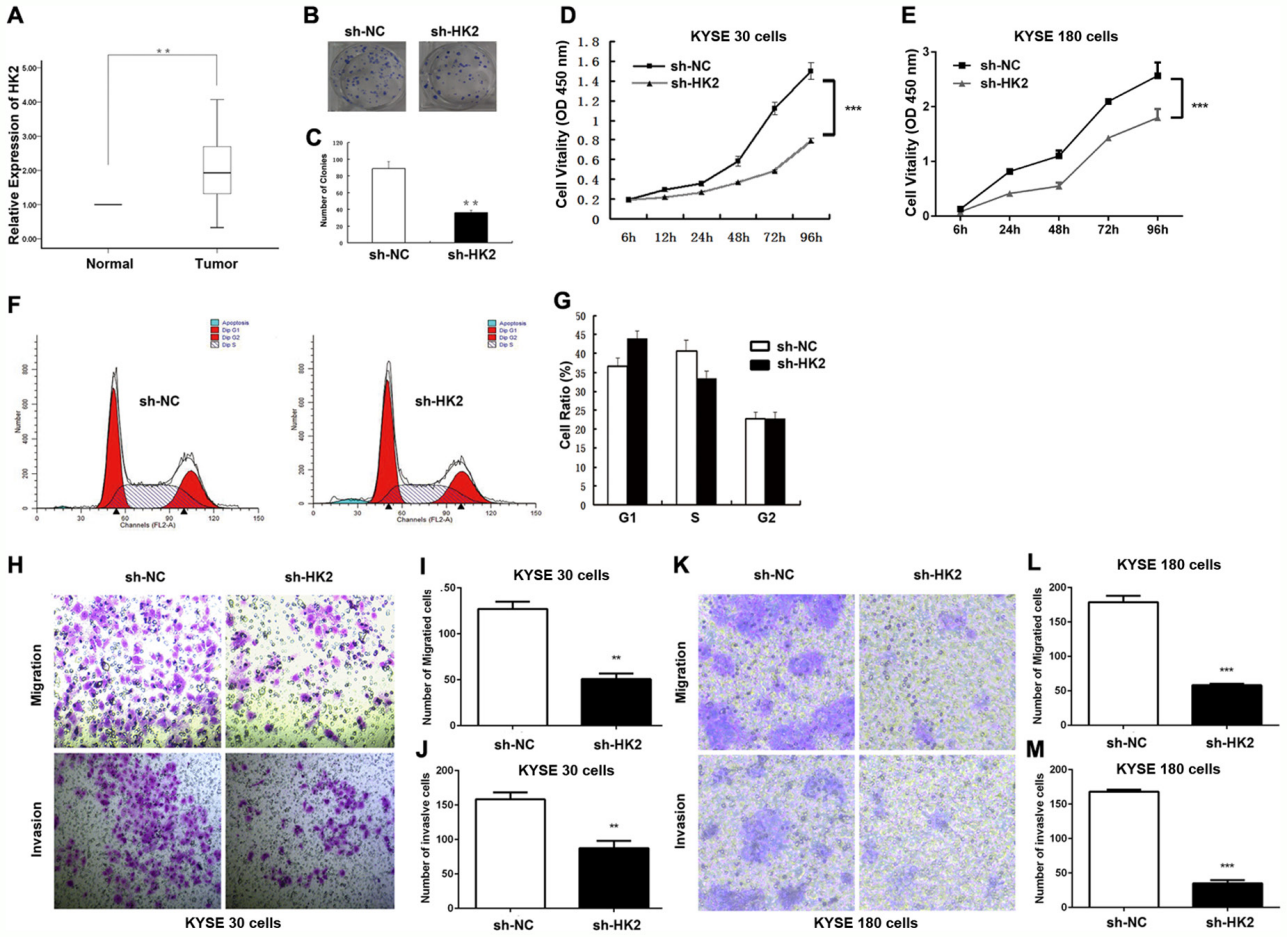
HK2 promoted cell proliferation, invasion and migration in ESCC Cells **(A)** ESCC patients with lymph node metastasis displayed significantly higher HK2 expression levels (*p* < 0.01). **(B, C)** Clone formation ability after HK2 depletion in KYSE30 cells. **(D, E)** Cell proliferation after HK2 depletion in KYSE30 cells (D) KYSE180 cells (E) were determined with CCK-8 assay. **(F, G)** Cell cycle distribution in KYSE30 cells was determined by flow cytometry using propidium iodide (PI) staining. **(H-M)** Transmembrane migration and invasion capacity were performed in KYSE30 cells (H, I, J) or KYSE180 (K, L, M) cells transfected with sh-NC or sh-HK2. Representative results from at least 3 independent experiments.

Reportedly, HK2 is overexpressed in tumours [39] and it is documented as a metabolic target for cancer therapy [10, 12, 40]. This also holds true with HK2 in ESCC tissues and their matched normal tissues, which showed HK2 was upregulated in ESCC tissues (Figure 4A).

We next evaluated whether HK2 modulates colony formation and proliferation in ESCC, by performing a colony formation assay. As shown in Figure 4B, the colonies of the shRNA-HK2 (sh-HK2) cells were smaller and fewer than those formed by the sh-NC cells. The number of colonies produced by the sh-HK2 cells was reduced by 59.6%. (*p* < 0.05, Figure 4C). Meanwhile, the CCK-8 assay revealed the optical density (OD) value at 450 nm for KYSE30 ESCC cells and KYSE180 ESCC cells treated with sh-HK2 was significantly lower from 48 h to 96 h (Figure 4D and 4E).

We next focused on the cell cycle. Flow cytometry (FCM) using propidium iodide (PI) staining was used to assess whether HK2 modulated cell cycle distribution. Knockdown of HK2 resulted in G0/G1 cell cycle arrest and reduction of S phase cells in KYSE30 ESCC cells (Figure 4F and 4G).

The effects of HK2 on the migratory and invasive behaviour of ESCC cell lines were assessed. HK2-silenced cells demonstrated a significantly lower transmembrane migration capacity compared with those transfected with sh-NC (Figure 4H and 4K). The migration activity was dramatically reduced by approximately 60.4% in sh-HK2 KYSE30 cells (Figure 4I) and 65.1% in KYSE180 cells (Figure 4L). Moreover, the invasion assay also indicated that the suppression of HK2 with sh-HK2 decreased invasion into the Matrigel substrate (Figure 4H and 4K). As shown in Figure 4J and 4M, sh-HK2 reduced the invasion by 44.2% in KYSE30 cells and 78.4% in KYSE180 cells. These results clearly demonstrate the importance of HK2 in promoting ESCC cell proliferation, invasion and migration.

### The HOTAIR/miR-125 and miR-143/HK2 axis in cell proliferation, invasion and migration of ESCC cells

**Figure 5:**
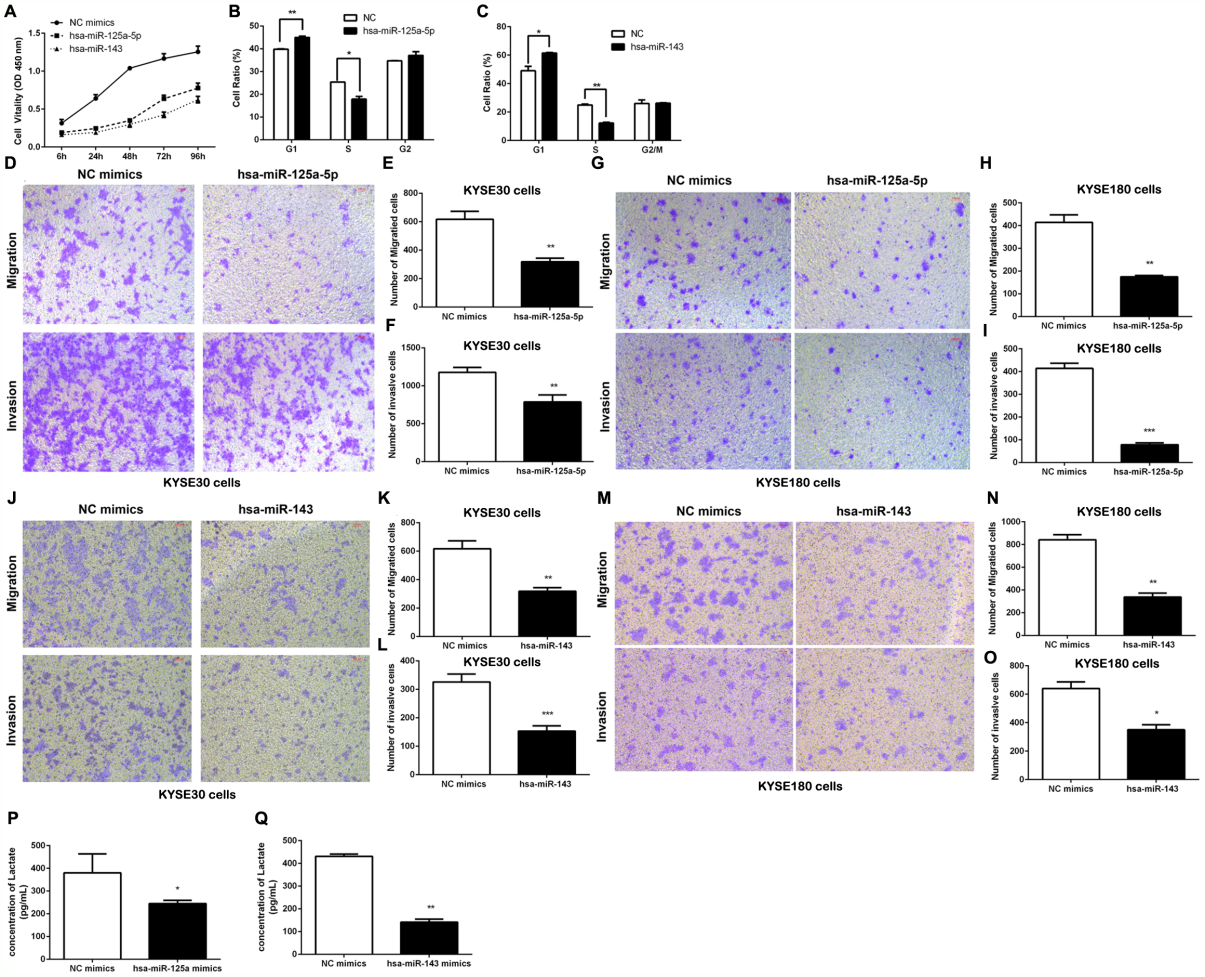
The effect of the HOTAIR/miR-125 and miR-143/HK2 axis on cell proliferation, invasion and migration in ESCC Cells **(A)** Cell proliferation in KYSE30 cells was determined with CCK-8 assay. **(B, C)** Cell cycle distribution after transfecting hsa-miR-125a-5p mimics (B) or hsa-miR-143 mimics (C) into KYSE30 cells was determined by flow cytometry using propidium iodide (PI) staining. **(D-I)** The transmembrane migration and invasion capacity in KYSE30 cells (D, E, F) or KYSE180 cells (G, H, I) infected with hsa-miR-125a-5p or sh-NC. **(J-O)** The transmembrane migration and invasion capacity of KYSE30 cells (J, K, L) or KYSE180 cells (M, N, O) infected with hsa-miR-143 or sh-NC. **(P, Q)** Lactate production in the culture media of hsa-miR-125a-5p-transfected (P) or hsa-miR-143-transfected (Q) KYSE30 cells was detected using metabolic assays. Representative results from at least 3 independent experiments.

The aforementioned data showed that HK2 promoted cell proliferation, invasion and migration in ESCC cells. We next wanted to pinpoint the underlying mechanism of the HOTAIR/miR-125 and miR-143/HK2 axis in the ESCC malignancy process. Initially, compared to the control group, the cell proliferation of miR-125 and miR-143 were dramatically lower than that of the control group from 24 h to 96 h (Figure 5A). Flow cytometry analyses indicated a significant reduction of S phase cells and an increase of cells in G1 arrest in the KYSE30 cells (Figure 5B and 5C). In the cell migration and invasion analysis, overexpression of miR-125 mimics and miR-143 mimics separately decreased migration and invasion in KYSE30 cells and KYSE180 cells (Figure 5D-5I).

The Warburg effect is a well-accepted phenomenon in which most cancer cells primarily utilize aerobic glycolysis for their energy needs even under normal oxygen concentrations [7]. Given the importance of HK2 in reprogramming glucose metabolism in cancer cells, we were interested in whether glucose metabolism was regulated by miR-125 and miR-143. We next transfected KYSE30 cells with either miR-125 or miR-143, and cell supernatants were harvested for lactate production analysis. As shown in Figure 5P and 5Q, we noted an obvious decrease in lactate concentration both in the miR-125 and the miR-143 group. These results indicate that activation of glucose metabolism by miR-125 and miR-143 targeting of HK2 is specific to ESCC.

**Figure 6:**
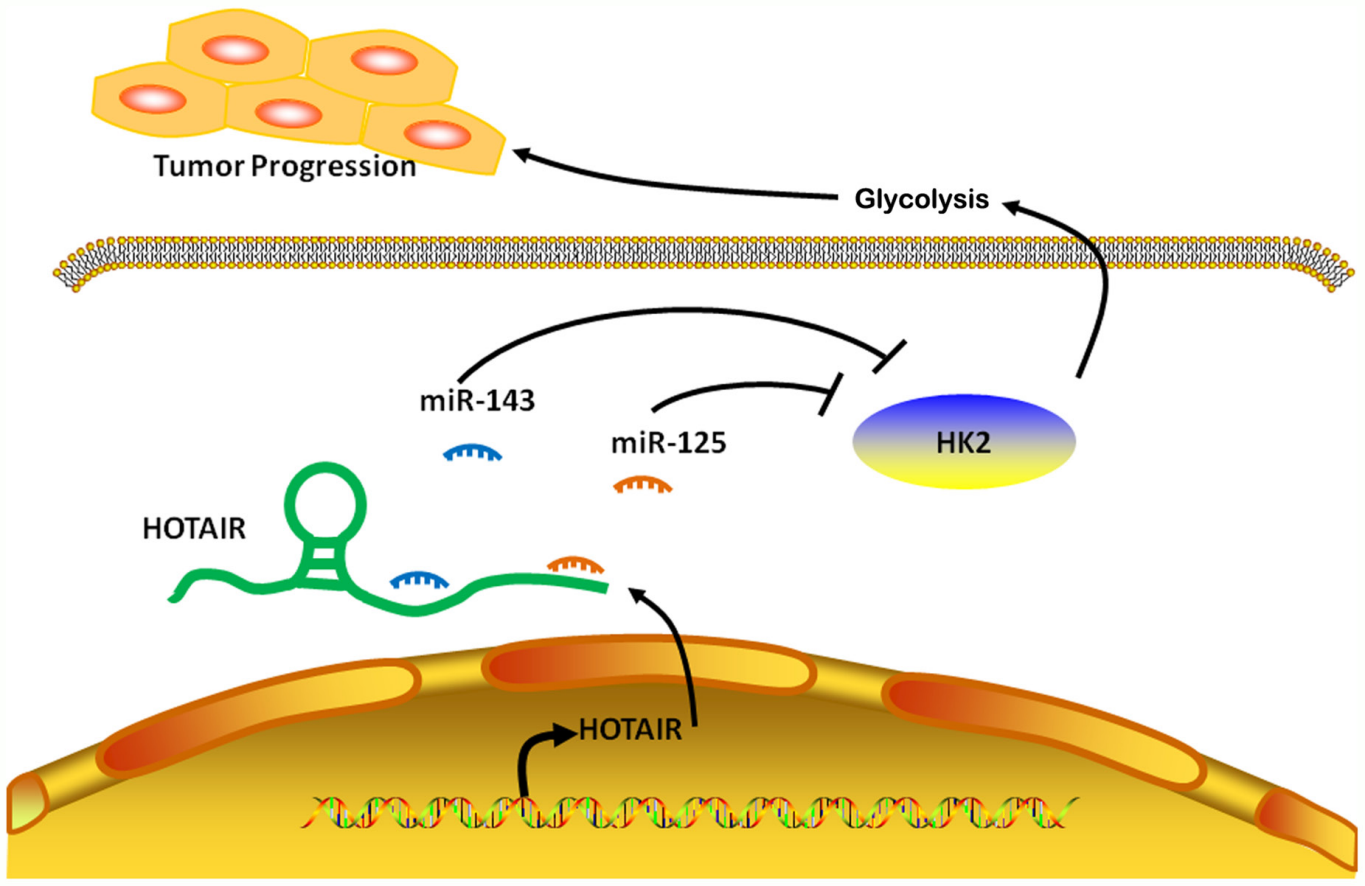
Model for regulation of HK2 by HOTAIR, acting as a ceRNA in ESCC In ESCC cells, lncRNA HOTAIR promotes HK2 expression by competitively binding miR-125 and miR-143, leading to the increased glycolysis, thus forming a tumor progression.

To summarize, a ceRNA model was proposed to summarize the HOTAIR/miR-125 and miR-143/HK2 pathway (Figure 6). HOTAIR negatively regulated miR-125 and miR-143 in ESCC through binding to miR-125 and miR-143 directly. In addition, miR-125 and miR-143 targeted HK2 and negatively regulated HK2 expression, indicating that HOTAIR influences the expression of HK2 in ESCC through miR-125 and miR-143.

## DISCUSSION

HOTAIR is a well-known molecule in the lncRNA world due to its involvement in reprogramming chromatin organization and promoting cancer cell metastasis [29–31]. An increasing amount of literature indicates that HOTAIR is highly expressed in several types of malignancies [41–43]. In our previous study, we investigated the biological role of HOTAIR in ESCC progression and its clinical significance, and we found that HOTAIR promotes the invasiveness and migration of ESCC cells and dramatically decreases the response of ESCC cells to apoptosis induction *in vitro*. Here, we determined *in vivo* that sh-HOTAIR reduced tumour growth compared to the control group. This demonstrated that HOTAIR can promote ESCC progression, which is in line with the *in vitro* results.

Inspired by the observation that HOTAIR is dysregulated during ESCC progression both *in vitro* and *in vivo*, we investigated the detailed molecular biological mechanism of how HOTAIR regulates ESCC progression through a ceRNA regulatory network. An outline of the ceRNA hypothesis attributes new and potentially predictable functionality to the coding and non-coding transcriptome, including its logic, and discusses recent experimental evidence supporting it and the consequences of altering its homeostasis. Overall, they hypothesize that all types of RNA transcripts (messenger RNAs, transcribed pseudogenes, and long non-coding RNAs) communicate through a new “language” mediated by microRNA binding sites (MREs or “microRNA response elements”) and that recent advances in experimental techniques are finally allowing us hear and translate this language [27].

The lncRNA-miRNA-mRNA crosstalk has been widely identified, where lncRNA competes for shared miRNA response elements, thus acting as a miRNA sponge that suppresses the binding of endogenous miRNAs to their target genes [44]. It has been shown that HOTAIR can control the expression of Rab 22a by sponging miR-373 in ovarian cancer [45], and modify miR-141 in regulating SKA2 in glioma [46]. In esophageal cancer, HOTAIR promotes the EMT and enhances metastasis by sponging miRNA-148a to regulate Sail2 [47]. These studies imply a very close interaction between lncRNA and miRNA. By using the Refseq, UCSC, GEO and miRbase databases, combined with RegRNA and PI TA bioinformatics software, we predicted and screened potential miRNA targets of HOTAIR. In our present study, whether HOTAIR functions as a ceRNA by directly interacting with miRNA was investigated. As shown by dual-luciferase reporter assays, miR-125 and miR-143 were able to bind to the 3’UTR of HOTAIR. miRNAs interact with the many proteins of the RISC. Among these, Argonaute (Ago) proteins bind to mature miRNAs to promote their binding to mRNA, thereby playing a central role in RNA silencing. A RIP assay confirmed that HOTAIR was enriched in Ago2-containing beads compared to controls, which was demonstrated by the interaction between HOTAIR and miR-125/miR-143.

This finding confirmed that the 3'UTR of HK2 mRNA constitutes a direct target of a miR-143 conserved sequence region. Simultaneously, miR-143 positively regulates HK2 protein expression at the post-transcriptional level in breast cancer. Inhibiting the expression of HK2 can inhibit the proliferation and survival of tumour cells in the breast cancer cell line MDA-MB-231 [48]. Interestingly, miR-143 can also inhibit HK2 expression in human lung cancer and neck squamous cell carcinoma (HNSCC) through targeting HK2 [49, 50]. As an oncogene, HK2 plays a major role in aerobic glycolysis, catalysing its first step and preventing glucose from entering the cell [10]. Previous studies have confirmed that HK2 expression was significantly higher in a variety of malignant tumours [10, 51]. HK2 is the primary hexokinase isoform in normal oesophageal squamous epithelium and in ESCC [52]. In zinc-deficiency (ZD)-associated esophageal neoplasia, ZD modulats miR-143 expression and its target HK2 [53]. Here, we further demonstrate that miR-125 and miR-143 can repress lactate production. These results reveal that HK2 binding to miR-125 and miR-143 leads to the regulation of lactic acid.

In conclusion, we focused on HOTAIR down-regulation of HK2 by sequestering the endogenous RNA miR-125 and miR-143, which impaired the balance between ESCC cell death and survival. Briefly, the present study suggests that glycolysis is involved in HOTAIR/miR-125- and miR-143/HK2-mediated tumour activation. For the first time, a ceRNA model of the HOTAIR/miR-125 and miR-143/HK2 regulatory axis was elucidated, in which HOTAIR acts as a competing endogenous RNA by sponging miR-125/miR-143 to promote HK2 expression, thereby facilitating the tumourigenesis of ESCC *in vitro* and *in vivo*. Investigation of this HOTAIR/miR-125 and miR-143/HK2 pathway may contribute to a better understanding of ESCC pathogenesis, and the components of this pathway are proposed to be good targets for clinical prevention and treatment of ESCC.

## MATERIALS AND METHODS

### Tissue samples and patient data

A total of 78 ESCC samples were obtained from patients undergoing surgery at the Department of Surgical Oncology, Affiliated Nanjing First Hospital of Nanjing Medical University between 2001 and 2008. The patients informations were described previously [31]. Briefly, All specimens were immediately frozen in liquid nitrogen and stored at -80°C until RNA extraction. No patient received chemotherapy or radiotherapy prior to surgery. The medical ethics committee of the Nanjing Hospital affiliated with the Nanjing Medical University approved the study.

### Cell lines and cell culture

The human ESCC cell lines KYSE30 and KYSE180 were established from samples extracted from human ESCC patients by Dr. Shimada [54, 55]. Cells were maintained in RPMI-1640 containing 10% FBS with 100 units/ml penicillin and 100 mg/ml streptomycin. These cells were cultured in a humidified 5% CO_2_ incubator at 37°C.

### shRNAs

To construct sh-HOTAIR and sh-HK2, target sequences were inserted into an RNAi-Ready pSIREN-RetroQ-ZsGreen vector. The shRNA sequences are shown below: sh-HOTAIR1, 5’-GAGACACATGGGTAACCTA-3’; sh-HOTAIR2, 5’-CTGCAACCTAAACCAGCAA-3’; and sh-HK2, 5’-GGTTGACCAGTATCTCTAC-3’.

### RNA isolation, reverse transcription, and qRT-PCR

Total RNA was extracted from cancerous / noncancerous specimens or cell lines using TRIzol reagent (Invitrogen, CA, USA). RNA was reverse transcribed into cDNA using a Prime-Script™ one step RT-PCR kit (TaKaRa, Dalian, China). The HOTAIR expression level was determined by qRT-PCR using the following primer sequences: 5’-CAGTGGGGAACTCTGACTCG-3’ (forward) and 5’-GTGCCTGGTGCTCTCTTACC-3’ (reverse). The primers of the HK2 gene were 5’-CAACTTCCGTGTGCTTTGGG-3’ (forward) and 5’-CAACGTCTCTGCCTTCCACT-3’ (reverse). Glyceraldehyde 3-phosphate dehydrogenase (GAPDH) was used as an internal control. The HOTAIR levels were normalized to GAPDH using the following primers: (forward) 5’-GTCAACGGATTTGGTCTGTATT-3’ and (reverse) 5’-AGTCTTCTGGGTGGCAGTGAT-3’. qRT-PCR reactions were performed using an ABI7500 System (Applied Biosystems, CA, USA) and SYBR Green PCR Master Mix (TaKaRa, Dalian, China).

### Western blotting analysis

The KYSE30 cells were transfected with sh-HOTAIR, sh-HK2, hsa-miR-125a-5p mimics or hsa-miR143 mimics (or sh-NC and NC mimics). At 48 h post-transfection, cell lysates were subjected to SDS-PAGE and transferred onto a PVDF membrane for western blotting. The following primary antibodies were used for western blotting: anti-human Actin polyclonal Ab (Proteintech, USA), anti-HK2 (Cell Signaling Technology, USA).

### Xenograft mouse model (the subcutaneous and orthotopic implanted models)

Sixteen, 4-week-old, male BALB/c nude mice weighing between 18 and 21 g were maintained under specific pathogen-free conditions. Then, 4 × 10^6^ KYSE30 cells transfected with sh-HOTAIR or negative control were injected subcutaneously into the right flank of the BALB/c nude mice (eight mice per group). Tumour volumes were examined every 3 days when the implants began to visibly increase in size. After 4 weeks, the mice were sacrificed, and the tumour volumes were calculated. All of the mouse experimental procedures were performed in accordance with the guidelines of the Institutional Animal Care and Use Committee. The protocol was approved by the Committee on the Ethics of Animal Experiments of Nanjing Hospital affiliated with the Nanjing Medical University. All surgeries was performed under chloral hydrate anaesthesia, and all efforts were made to minimize suffering.

### Plate colony formation assay

KYSE30 cells were transfected with 50 nM sh-HOTAIR, sh-HK2 or sh-NC. Cells were harvested after 24 h and seeded into a 6-well plate (500 cells/well, 2 wells/group). After incubation at 37°C and 5% CO_2_ for 14 days, the cells were washed twice with PBS and stained with Crystal Violet Staining Solution. The colonies containing ≥ 50 cells were counted under a microscope, and the plate clone formation efficiency was calculated using the following formula: plate clone formation efficiency = (number of colonies / number of cells inoculated) × 100%.

### Cell proliferation assay

KYSE30 and KYSE180 cells were transfected with sh-HOTAIR, sh-HK2, sh-NC, hsa-miR125a-5p mimics, hsa-miR-143 mimics or NC mimics. Transfected cells were harvested after 24 h, seeded in a 96-well plate (1,000 cells/200 μl/well) and cultured in a humidified 37°C and 5% CO_2_ atmosphere. The Cell Counting Kit-8(CCK8, Dojindo, Tokyo, Japan) was used to measure cell growth following the manufacturer’s instructions. Absorbance values were determined at 450 nm using a microplate reader. Experiments were performed in triplicate.

### Flow cytometry

KYSE30 and KYSE180 cells were plated into 6-well plates (2 x 10^5^ cells/well) in antibiotic-free medium after being transfected with sh-HOTAIR, sh-HK2, sh-NC, hsa-miR125a-5p mimics, hsa-miR-143 mimics or NC mimics for 48 h. Cells were collected for apoptosis analysis, washed twice and stained with fluorescein isothiocyanate (FITC)-Annexin V and propidium iodide (PI, BD Bioscience) using a FITC Annexin V Apoptosis Detection Kit (BD Biosciences) according to the manufacturer’s manual. Apoptotic cells were analysed using flow cytometry (CYTOMICS FC 500, Beckman Coulter, Miami, FL). We calculated the apoptotic cells according to the manufacturer's instructions as follows: the fraction of cells in the upper right quadrant (representing late apoptotic cells) and the fraction of cells in the lower right quadrant (representing early apoptotic cells).

The KYSE30 cells for cell cycle analysis were suspended in 70% chilled ethanol and washed with cold PBS prior to staining with PI. Cell cycle distribution was measured using flow cytometry (CYTOMICS FC 500, Beckman Coulter, Miami, FL). The percentage of cells in the G0-G1, S, and G2-M phases were counted and compared. All experiments were conducted in triplicate.

### RNA-binding protein immunoprecipitation (RIP) assay

RNA immunoprecipitation was performed using an EZMagna RIP kit (Millipore, Billerica, MA, USA) following the manufacturer’s protocol. KYSE30 cells at 80-90% confluency were scraped off of the plates and then lysed in complete RIP lysis buffer, after which 100 μl of whole cell extract was incubated with RIP buffer containing magnetic beads conjugated with human anti-Ago2 antibody (Millipore); the negative control was normal mouse IgG (Millipore). Samples were incubated with Proteinase K with shaking to digest the protein, and then immunoprecipitated RNA was isolated. The RNA concentration was measured using a NanoDrop spectrophotometer (Thermo Scientific), and the RNA quality was assessed using a bioanalyser (Agilent, Santa Clara, CA, USA). Furthermore, purified RNA was subjected to qRT-PCR analysis using the respective primers to demonstrate the presence of the binding targets.

### *In vitro* cell migration and invasion assays

KYSE30 and KYSE180 cells were transfected with shRNAs targeting HOTAIR, HK2, hsa-miR-125a-5p mimics, hsa-miR-143 mimics or a scrambled negative control (sh-NC and NC mimics). At 24 h post-transfection, transfected cells were harvested and subjected to the following assays.

For migration assays, transfected cells (1×10^5^) were plated in the top chamber of Transwell assay inserts (Millipore, Billerica, MA) with a membrane containing 8-μm -diameter pores in 200 μl of serum-free RPMI-1640. Assays were conducted in triplicate. Inserts were then placed into the bottom chamber of a 24-well plate containing RPMI-1640 with 10% FBS as a chemoattractant. After 24 h of incubation, the remaining cells were removed from the top layer of the insert by scrubbing with a sterile cotton swab. Invading cells from the bottom surface were stained with 0.1% crystal violet prior to being examined, counted, and photographed using digital microscopy. Cells were counted in five random fields for each chamber, and the average value was calculated.

For invasion assays, transfected cells (4×10^5^) were plated in the top chamber with a Matrigel-coated membrane. The bottom chambers were filled with conditioned medium. After a 48 h incubation period, the number of migrated cells on the lower side of the membrane was counted as described previously.

### In silico analysis and dual-luciferase assay

Screening for miR-125a-5p- and miR-143-regulated genes and pathway analysis was performed using databases. The predicted target genes and their miRNA binding site seed regions were investigated using TargetScan (release 6.2, http://www.targetscan.org/). The sequences of the predicted mature miRNAs were confirmed using miRBase. To identify signalling pathways regulated by miR-125 and miR-143 in RCC, upregulated genes targeted by these miRNAs were analysed in terms of the Kyoto Encyclopedia of Genes and Genomes (KEGG) pathway categories using the GENECODIS program (http://genecodis.cnb.csic.es/). After being placed into 48-well plates, HEK 293T cells were cotransfected with pLMP-miR-let7g/130a/143/145*/203/125 or pLMP vector (200 ng), pRL-TK (2 ng, Promega), and luciferase reporter plasmids (50 ng) containing wild-type (W.T.) or mutant type (Mut) of the p27kip1 3’UTR using Lipofectamine 2000 (Invitrogen). Forty-eight hours after transfection, firefly and Renilla luciferase activities were measured by a Dual-Luciferase Reporter Assay System (Promega).

### Metabolic assays

KYSE30 cells were transfected with hsa-miR-125a-5p mimics, hsa-miR-143 mimics or a scrambled negative control (NC mimics). At 24 h post-transfection, the supernatants of the transfected cells were harvested and subjected to the following assays. Lactate production in the culture media of cells was detected using a lactate assay kit (Biovision, Milpitas, CA) according to the manufacturer’s instructions.

### Statistical analysis

Continuous data were analysed using an independent *t*-test between the two groups, whereas categorical data were analysed with a chi-square test. Overall survival curves were plotted according to the Kaplan-Meier method, with a log-rank test applied for comparison. Variables were used in multivariate analysis on the basis of the Cox proportional hazards model. All statistical analyses were performed using SPSS for Windows v.17.0 (SPSS, Chicago, IL). *p <* 0.05 was considered statistically significant.
