# Correction: HOTAIR regulates HK2 expression by binding endogenous miR-125 and miR-143 in oesophageal squamous cell carcinoma progression

**DOI:** 10.18632/oncotarget.25377

**Published:** 2018-05-04

**Authors:** Jian Ma, Yanxin Fan, Tingting Feng, Fangjun Chen, Zhipeng Xu, Suqing Li, Qingfeng Lin, Xiaoting He, Weihong Shi, Yang Liu, Zhihua Liu, Bin Zhu, Xiufeng Cao

**Affiliations:** ^1^ Department of Surgical Oncology, Affiliated Nanjing First Hospital of Nanjing Medical University, Oncology Center of Nanjing Medical University, Nanjing 210008, Jiangsu Province, China; ^2^ Jiangsu Cancer Hospital, Medical School of Nanjing University, Nanjing 210008, Jiangsu Province, China; ^3^ Institute of Biology and Medical Sciences, Jiangsu Key Laboratory of Infection and Immunity, Soochow University, Suzhou 215123, Jiangsu Province, China; ^4^ The Comprehensive Cancer Centre of Drum Tower Hospital, Medical School of Nanjing University, Clinical Cancer Institute of Nanjing University, Nanjing 210008, Jiangsu Province, China; ^5^ Yancheng Vocational Institute of Health Sciences, Yancheng 224005, Jiangsu Province, China; ^6^ Department of Pharmacy, No.401 Hospital of Chinese People's Liberation Army, Qingdao 266071, Shandong Province, China; ^7^ State Key Laboratory of Molecular Oncology, National Cancer Center/Cancer Hospital, Chinese Academy of Medical Sciences, Beijing 100021, China

**This article has been corrected:** The correct order of Author names are given below:

Jian Ma^1,*^, Yanxin Fan^2,*^, Tingting Feng^1,3,*^, Fangjun Chen^4^, Zhipeng Xu^1^, Suqing Li^1^, Qingfeng Lin^1^, Xiaoting He^1^, Weihong Shi^1,5^, Yang Liu^6^, Zhihua Liu^7^, Bin Zhu^1^ and Xiufeng Cao^1^

Original article: Oncotarget. 2017; 8:86410-86422. https://doi.org/10.18632/oncotarget.21195

