# Correction: HOTAIR regulates HK2 expression by binding endogenous miR-125 and miR-143 in oesophageal squamous cell carcinoma progression

**DOI:** 10.18632/oncotarget.26801

**Published:** 2019-03-12

**Authors:** Jian Ma, Yanxin Fan, Tingting Feng, Fangjun Chen, Zhipeng Xu, Suqing Li, Qingfeng Lin, Xiaoting He, Weihong Shi, Yang Liu, Zhihua Liu, Bin Zhu, Xiufeng Cao

**Affiliations:** ^1^ Department of Surgical Oncology, Nanjing First Hospital, Nanjing Medical University, Nanjing 210008, Jiangsu Province, China; ^2^ Jiangsu Cancer Hospital, Medical School of Nanjing University, Nanjing 210008, Jiangsu Province, China; ^3^ Institute of Biology and Medical Sciences, Jiangsu Key Laboratory of Infection and Immunity, Soochow University, Suzhou 215123, Jiangsu Province, China; ^4^ The Comprehensive Cancer Centre of Drum Tower Hospital, Medical School of Nanjing University, Clinical Cancer Institute of Nanjing University, Nanjing 210008, Jiangsu Province, China; ^5^ Yancheng Vocational Institute of Health Sciences, Yancheng 224005, Jiangsu Province, China; ^6^ Department of Pharmacy, No.401 Hospital of Chinese People’s Liberation Army, Qingdao 266071, Shandong Province, China; ^7^ State Key Laboratory of Molecular Oncology, National Cancer Center/Cancer Hospital, Chinese Academy of Medical Sciences, Beijing 100021, China

**This article has been corrected:** The correct affiliation information is given below:

^**1**^ Department of Surgical Oncology, Nanjing First Hospital, Nanjing Medical University, Nanjing 210008, Jiangsu Province, China

Original article: Oncotarget. 2017; 8:86410-86422. https://doi.org/10.18632/oncotarget.21195

